# Disruption of winter influenza activity in Wuxi, China during and after the COVID-19 pandemic (2013–2025): a counterfactual time-series analysis

**DOI:** 10.3389/fpubh.2026.1851342

**Published:** 2026-06-18

**Authors:** Miao Wang, Yan Wang, Chao Shi, Yumeng Gao, Shuo Liu, Yuan Shen

**Affiliations:** 1 School of Public Health, Nanjing Medical University, Nanjing, China; 2 The Affiliated Wuxi Center for Disease Control and Prevention of Nanjing Medical University, Wuxi Center for Disease Control and Prevention, Wuxi, China

**Keywords:** counterfactual analysis, COVID-19, epidemic intensity, epidemic timing, seasonal influenza

## Abstract

**Background:**

The COVID-19 pandemic and associated non-pharmaceutical interventions (NPIs) altered the circulation of respiratory viruses, and influenza activity declined worldwide during 2020–2022. However, the magnitude of these disruptions and their post-pandemic effects on epidemic dynamics remain insufficiently characterized.

**Methods:**

We analyzed weekly influenza surveillance data from 2013 to 2025 in Wuxi, eastern China. We constructed a composite influenza activity index using principal component analysis (PCA). We then trained a Bayesian structural time series (BSTS) model on pre-pandemic (2013–2019) values of this index to generate counterfactual estimates of influenza activity. We classified epidemic intensity using the Moving Epidemic Method (MEM) and quantified epidemic timing, including onset, end, and duration, using a modified Maximum Curvature Method (MCM) for both observed and counterfactual data.

**Results:**

Influenza activity was substantially suppressed during the COVID-19 pandemic (2020–2022), characterized by a near-complete interruption of transmission in the 2020/2021 season. During the subsequent post-pandemic period (2023–2025), influenza activity rebounded relative to counterfactual projections, although recovery trajectories varied across seasons. Overall, the trajectory of influenza activity was marked by a sharp decline during the pandemic, followed by a partial and heterogeneous recovery. MEM-based analysis indicated reduced epidemic intensity during the pandemic, while observed peak activity exceeded counterfactual estimates in some post-pandemic seasons. MCM-based analysis showed that peak timing remained broadly stable overall, although both delayed and advanced peaks were observed across seasons, particularly during the pandemic and transition period. Epidemics were generally shorter and ended earlier during the early pandemic, whereas post-pandemic seasons exhibited heterogeneous patterns in timing and duration with no consistent directional trends across seasons.

**Conclusions:**

Influenza activity was markedly suppressed during the COVID-19 pandemic, reflecting disrupted transmission dynamics. The subsequent rebound, accompanied by heterogeneous epidemic patterns, suggests alterations in influenza seasonal dynamics and highlights the need for adaptive surveillance and response strategies.

## 1 Introduction

Seasonal influenza remains a major cause of respiratory morbidity and mortality worldwide and continues to place pressure on healthcare systems each year (1). Influenza activity exhibits pronounced seasonal patterns driven by climate, population contact patterns, viral evolution, and population immunity (2). In China, the seasonality of influenza activity varies geographically. Southern coastal regions typically experience semi-annual or summer-dominant activity, while temperate and temperate-subtropical regions generally exhibit winter-spring epidemics. In the Yangtze River Delta region, including Wuxi, influenza activity has generally followed a winter-spring seasonal pattern prior to the COVID-19 pandemic (3–5).

The COVID-19 pandemic emerged in early 2020 and led to the widespread use of non-pharmaceutical interventions (NPIs) worldwide. In China, stringent control measures were implemented during the early pandemic phase and were later gradually relaxed. The “zero-COVID” policy ended during the Omicron wave in late 2022 (6, 7). These interventions altered population mobility, social contact patterns, and healthcare-seeking behaviors. Although primarily intended to reduce transmission of severe acute respiratory syndrome coronavirus 2 (SARS-CoV-2), NPIs also affected the circulation of other respiratory viruses, including influenza (8).

Multiple countries reported a marked reduction in influenza activity during the early phase of the pandemic (8, 9). Following the relaxation of NPIs, influenza activity re-emerged with heterogeneity across regions. Some areas reported delayed epidemics, whereas others reported stronger outbreaks than expected (10, 11). However, most previous studies have primarily compared influenza activity before and after the pandemic or described short-term trends (12). While these approaches confirm substantial changes in influenza activity, they provide limited ability to quantify how influenza dynamics might have evolved in the absence of the COVID-19 pandemic. Without an explicit counterfactual baseline, it is difficult to quantify the magnitude and duration of pandemic-associated disruptions. Influenza surveillance indicators may also be influenced by changes in healthcare utilization and testing practices during major public health events (8, 13). For example, influenza cases from the national surveillance system reflect clinically diagnosed disease burden, whereas positivity rate from sentinel laboratories provides virological evidence of infection. Each indicator captures a distinct dimension of influenza activity, yet neither alone fully represents transmission dynamics. Because these indicators are correlated, integrating them into a composite measure may provide a more stable and robust representation of influenza activity. Principal component analysis (PCA) is widely used to extract dominant shared variation from correlated surveillance indicators and to reduce measurement noise (14, 15).

In addition, previous studies have focused primarily on epidemic magnitude, whereas changes in temporal structure, including epidemic onset, duration, and peak timing, remained less systematically characterized. Wuxi represents an optimal setting for addressing these limitations, owing to its stable, winter-dominant influenza seasonality and comprehensive, long-term surveillance system. The availability of weekly surveillance data spanning 2013–2025 facilitates the establishment of a pre-pandemic baseline, the characterization of pandemic-period dynamics, and the assessment of post-pandemic recovery trajectories.

To address these gaps, we analyzed weekly influenza surveillance data from Wuxi to evaluate changes in winter-dominant influenza activity during and after the COVID-19 pandemic. We constructed a composite influenza activity index by integrating laboratory-confirmed cases and positivity rate using PCA. To estimate influenza activity in the absence of the pandemic, we applied a Bayesian structural time series (BSTS) model. This approach captures long-term trends and seasonal patterns to generate counterfactual estimates of influenza activity under a hypothetical no-pandemic scenario. We assessed epidemic intensity using the Moving Epidemic Method (MEM), a method widely applied in influenza surveillance systems to define epidemic thresholds and classify seasonal intensity. To characterize the timing of epidemics, we estimated onset, end, and duration using a modified version of the Maximum Curvature Method (MCM). This method identifies the onset and end of an epidemic based on changes in epidemic growth patterns. Together, these approaches enabled a multidimensional characterization of pandemic-associated changes in influenza activity, intensity, and temporal structure.

## 2 Materials and methods

### 2.1 Data source

We obtained weekly reports of laboratory-confirmed influenza cases from the National Infectious Disease Surveillance System (NIDSS) in China from January 2013 to December 2025. The NIDSS is an internet-based timely reporting system that was developed by the Chinese Center for Disease Control and Prevention and officially launched in 2004 (16, 17).

Influenza-like illness (ILI) and virological surveillance data were obtained from the Wuxi Influenza Surveillance Network, which comprises four sentinel hospitals. Each sentinel hospital registered outpatient cases of ILI (body temperature ≥ 38 °C with either cough or sore throat in the absence of an alternative diagnosis) and collected at least 20 respiratory specimens per week from ILI outpatients. Influenza viruses were detected and subtyped using reverse transcription polymerase chain reaction (RT-PCR). All ILI data and laboratory results were electronically submitted to the web-based National Influenza Surveillance Information System. Based on these data, the weekly positivity rate was defined as the proportion of respiratory specimens from ILI outpatients testing positive for influenza (18, 19).

**Figure 1:**
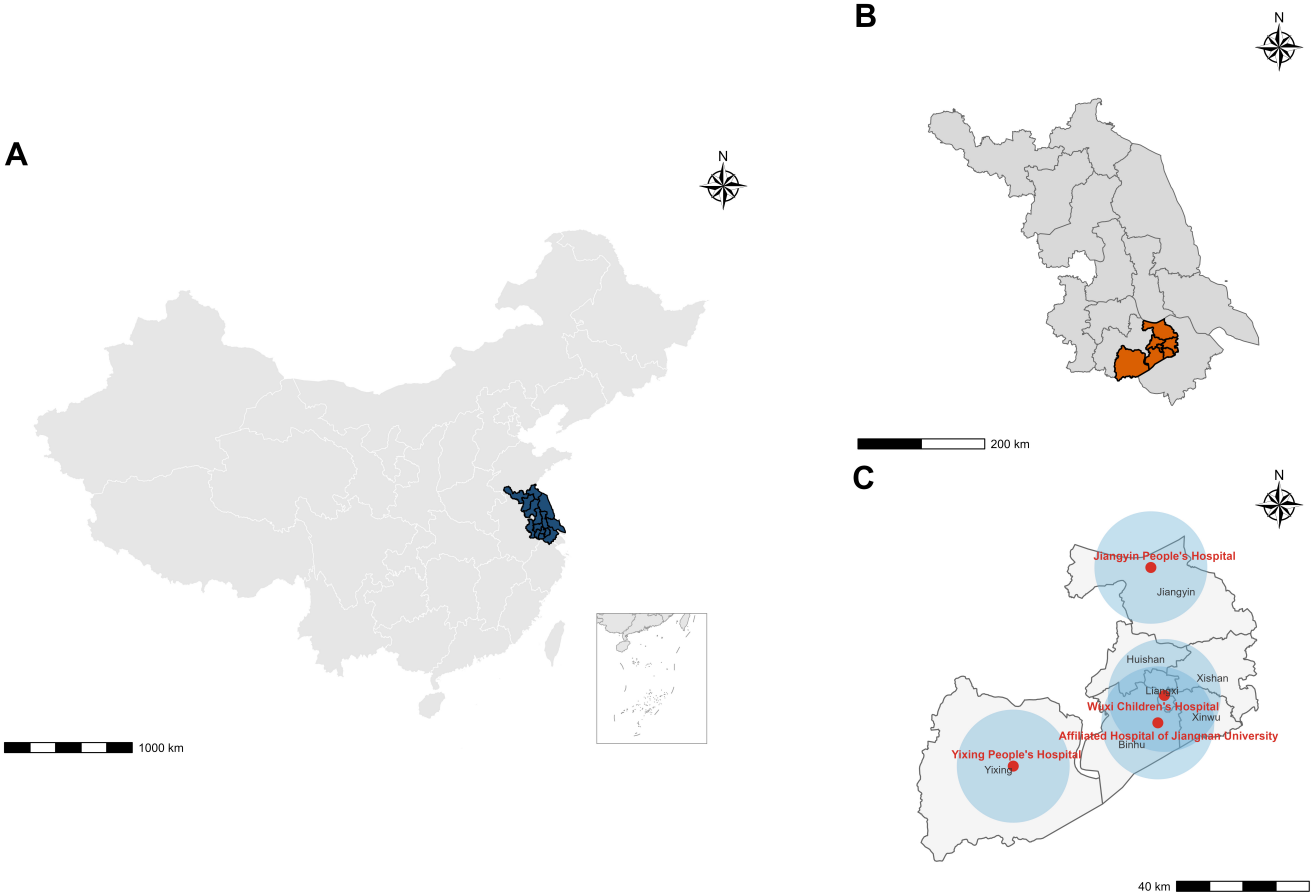
Geographic location of Wuxi and spatial distribution of the sentinel influenza surveillance system. **(A)** Location of Jiangsu Province within China. **(B)** Location of Wuxi city within Jiangsu Province. **(C)** Geographic distribution of the four participating sentinel hospitals within Wuxi municipality. Shaded circles provide a schematic visualization of the approximate surveillance service regions and do not represent formally defined hospital catchment boundaries. Administrative district boundaries are shown for geographic context.

Population data for Wuxi and its administrative divisions were obtained from the *Wuxi Statistical Yearbook 2025*. Detailed population statistics and the geographic distribution of sentinel hospitals are provided in Supplementary Table S1 and Figure 1.

The winter influenza season was defined as epidemiological week 40 of a given year through week 20 of the following year (20). The period 2013–2019 was defined as the pre-pandemic baseline, serving as the training dataset for model development. The pandemic period was defined as beginning in January 2020; accordingly, data from 2020 to 2022 constitute the pandemic period, which was characterized by the widespread implementation of non-pharmaceutical interventions (NPIs). Data from 2023 onward are defined as the post-pandemic transition period.

### 2.2 Statistical analysis

All analyses were conducted in R (version 4.3.2). The analytical framework integrated both inferential and descriptive methodologies. Bayesian inference was exclusively implemented via the BSTS model to estimate counterfactual influenza activity and quantify pandemic-associated effects, with results expressed through posterior distributions and credible intervals (CrIs). In contrast, PCA, the MEM, and the modified MCM served as descriptive tools for data integration and epidemic characterization. Specifically, PCA was used to construct a composite influenza activity index, while the MEM and MCM were applied to characterize epidemic intensity and temporal structure, respectively. Notably, these descriptive procedures were not intended for hypothesis testing or probabilistic inference. PCA was implemented using base R functions. Counterfactual modeling based on BSTS was performed using the “bsts” package (version 0.9.10). Epidemic thresholds and intensity categories were derived using the “mem” package (version 2.19).

#### 2.2.1 Composite influenza activity index

We constructed a composite influenza activity index by integrating multiple surveillance indicators, aiming to obtain a robust measure of influenza activity. To reduce skewness, we log-transformed weekly influenza cases using log(1 + cases); similarly, we smoothed weekly influenza positivity rate using a five-week centered moving average to mitigate short-term fluctuations. We then standardized both variables using Z-score normalization based on the pre-pandemic training period.

We performed PCA on the pre-pandemic data to capture the dominant shared variation among these indicators (14, 15). We retained the first principal component, which explained the largest proportion of variance, as the composite influenza activity index. When necessary, the orientation of the component was reversed so that larger index values consistently indicated higher influenza activity.

The explicit formulation and standardization procedure of the composite index are provided in the Supplementary Material S1.

#### 2.2.2 Counterfactual modeling using bayesian structural time series

We applied a Bayesian structural time series (BSTS) model to generate counterfactual estimates of influenza activity in the absence of the COVID-19 pandemic, using the composite index as the outcome variable (16, 21). The model was formulated as an additive state-space process consisting of a local level component (μ_*t*_) representing long-term trend dynamics and a seasonal component (τ_*t*_) with a 52-week periodicity capturing annual influenza seasonality.

The observation and state equations were defined as follows:

***Z*_*t*_** **=** **μ_*t*_** **+** **τ_*t*_** **+** **ε_*t*_** , **ε_*t*_** **~*N*** **(0**, **σ**^**2**^**ε)**

**μ**_*t***+1**_ **=** **μ_*t*_** **+** **η**_*t*_ , **η_*t*_** **~*N*** **(0**, **σ**^**2**^**η)**

**τ_*t*+1_*1*** **=** ***T*τ_*t*_** **+** **ξ**_*t*_ , **ξ**_*t*_ **~*N*** **(0**, **σ**^**2**^**ξ)**

We specified weakly informative default priors for the observation and state evolution variance parameters in the bsts package. We did not introduce any custom hyperparameters. In all model specifications, we defined *t* as epidemiological week.

We fitted the model using pre-pandemic data (2013–2019) to characterize baseline seasonal and trend dynamics. Based on the estimated state-space components, we generated counterfactual trajectories for 2020–2025 by forward extrapolation. We summarized weekly counterfactual values using posterior predictive means. For cumulative effect estimation (Section 2.2.3), we reported posterior medians together with 95% CrIs, as the posterior distributions showed mild skewness.

Posterior uncertainty increased progressively beyond 2022, consistent with the expanding forecast horizon in state-space models. We therefore restricted inferential interpretation of pandemic-associated effects to the 2020–2022 period, where the 95% CrI for cumulative effects remained entirely below zero. We treated estimates for 2023–2025 as non-inferential forecasts rather than inferential results.

We estimated model parameters using Markov Chain Monte Carlo sampling with 5,000 iterations, discarding the first 1,000 iterations as burn-in. We assessed convergence and sampling adequacy using effective sample size diagnostics, posterior predictive checks, and sensitivity analyses under alternative model specifications and thinning settings (Supplementary Tables S2–S4).

#### 2.2.3 Quantification of pandemic effects

We quantified the impact of the COVID-19 pandemic on influenza activity by comparing observed values ($z_{t}^{obs}$) with the counterfactual estimates ($z_{t}^{cf}$).

The cumulative absolute effect (22) was defined as:


$$\begin{array}{l} Z_{t}=\mu_{t}+\tau_{t}+\varepsilon_{t}\varepsilon_{t}\sim N(0,\sigma^{2}\varepsilon )\;\\ \mu_{t+1}=\mu_{t}+\eta_{t}\eta_{t}\sim N(0,\sigma^{2}\eta )\;\\ \tau_{t+1}1=T\tau_{t}+\xi_{t},\xi_{t}\sim N(0,\sigma^{2}\xi ) \end{array}$$

representing the total net change in influenza activity during the study period.

The cumulative relative effect (22) was calculated as the ratio of the cumulative absolute effect to the cumulative counterfactual projections:


$$\sum_{t=t_{0}}^{T}\left(z_{t}^{obs}-z_{t}^{cf}\right)$$ (1)

This metric presents the relative magnitude of change in influenza activity as a percentage.

#### 2.2.4 Seasonal epidemic intensity classification using MEM

We assessed epidemic intensity using the MEM (23). We derived intensity thresholds from the distribution of peak values observed during pre-pandemic seasons (2013–2019). Specifically, we defined the epidemic threshold using the 40th percentile of historical peak values. We then defined intensity categories (low, medium, high, and very high) using standard MEM cutoffs based on the 50th, 90th, and 97.5th percentiles. These thresholds were subsequently used to descriptively classify observed seasonal epidemic intensity.

#### 2.2.5 Temporal structure analysis using the modified maximum curvature method

To characterize changes in the temporal structure of seasonal influenza epidemics, we applied a modified MCM (24) to determine the onset, end, and duration of each influenza season. Because the influenza activity indicator is a dimensionless PCA-derived index rather than an absolute measure, we used a season-specific adaptive threshold to ensure comparability across seasons. Specifically, we defined the threshold as the 75th percentile of the composite index values within each season, which represents a balanced choice within the commonly used 70–80% range, avoiding both premature epidemic detection at lower thresholds and delayed identification at higher thresholds.

Within each season, we estimated the local curvature of the epidemic curve using a sliding-window approach. We used directional information derived from the curve's geometric properties to distinguish ascending and descending phases of epidemic activity. We identified the weeks of epidemic onset and end as the points of maximum curvature that satisfied the directional criteria and remained below the seasonal threshold. We defined epidemic duration as the inclusive interval between onset and end.

To evaluate the robustness of the results, sensitivity analyses were conducted by varying the sliding-window length (3–7 weeks) and the percentile threshold (70–80%) (Supplementary Table S5). The estimated epidemic timing indicators were generally robust across parameter combinations, with end estimates showing greater stability than onset estimates. We report all weeks as relative weeks within the influenza season, where week 1 corresponds to epidemiological week 40 of the monitoring year. The detailed mathematical formulation, curvature computation, and full algorithmic workflow of the modified MCM are given in Supplementary Material S2.

## 3 Results

### 3.1 Overview of influenza activity and construction of the composite index

Across influenza seasons from 2012/2013 to 2024/2025, influenza activity in Wuxi exhibited a consistent winter-spring seasonal pattern, with peak timing typically occurring between epidemiological weeks 47 and 10 of the following year. Influenza activity also varied markedly across the pre-pandemic, pandemic, and post-pandemic phases.

During the pre-pandemic period (2012/2013–2018/2019), influenza activity remained at a moderate level, with a mean weekly case count of 17.7 (median: 9; peak: 133) and a mean positivity rate of 16.5% (median: 10.0%). Influenza activity declined sharply during the pandemic period (2019/2020–2021/2022), reaching its lowest level in the 2020/2021 season. Influenza activity rebounded during the post-pandemic period (2022/2023–2024/2025), with the highest weekly peak magnitude occurring in the 2022/2023 season.

The PCA showed that the first principal component alone explained 90.5% of the total variance, indicating strong shared variation between cases and positivity rate. Both indicators contributed positively to the composite index, with factor loadings of 0.719 and 0.695. The resulting composite index accurately captured observed seasonal fluctuations and provided a unified measure of influenza activity across the study period. Descriptive statistics are presented in Supplementary Table S6.

### 3.2 Counterfactual impact of the COVID-19 pandemic on influenza activity

#### 3.2.1 Counterfactual changes in overall influenza activity and cumulative magnitude based on the BSTS Model

**Figure 2:**
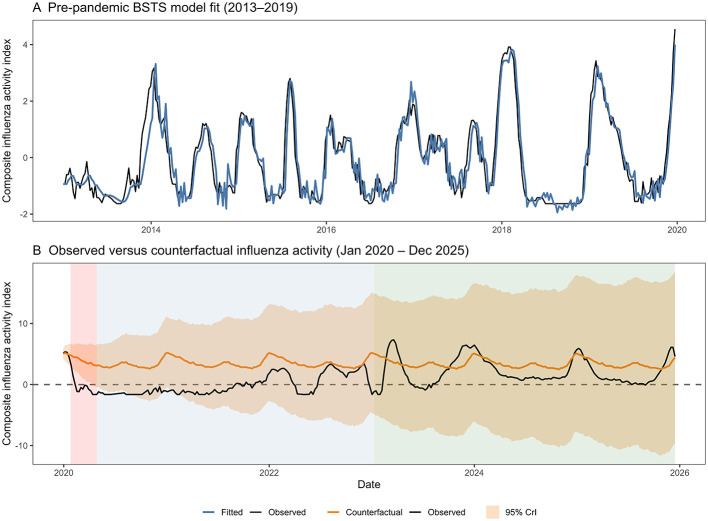
BSTS model fit and counterfactual influenza activity trajectories. **(A)** Pre-pandemic model fit (2013–2019) showing observed influenza activity (black line), posterior fitted means (blue line), and 95% CrIs (blue shaded region). **(B)** Observed influenza activity (black line) and BSTS-derived counterfactual trajectories (orange line) for Jan 2020–Dec 2025, with corresponding 95% CrIs (orange shaded region). Shaded background regions indicate different pandemic phases: early emergency response (red), sustained non-pharmaceutical intervention period (blue), and post-pandemic transition period (green).

The BSTS model demonstrated good fit during the pre-pandemic training period (*R*^2^ = 0.868, *RMSE* = 0.510, *MAE* = 0.377), with observed values closely tracking posterior fitted trajectories (Figure 2A). Descriptive statistics of the counterfactual composite influenza activity index are provided in Supplementary Table S7. Based on pre-2020 data, the model generated posterior predictive counterfactual estimates of influenza activity (95% CrI) under a no-pandemic scenario.

**Figure 3:**
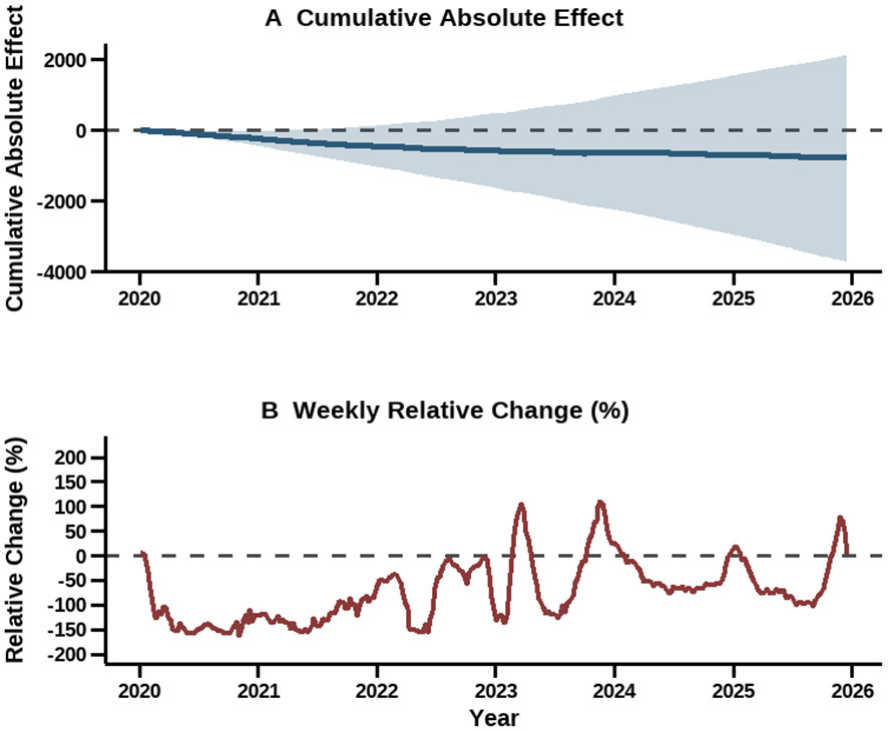
Counterfactual analysis of the impact of the COVID-19 pandemic on influenza activity. **(A)** Cumulative Absolute Effect: The cumulative absolute effect is defined as the accumulated difference between the observed composite influenza activity index and its counterfactual estimates under a no-pandemic scenario. The blue line shows the cumulative difference over time, and the shaded band represents the 95% CrI derived from the BSTS posterior distribution. The dashed horizontal line indicates zero difference, representing no deviation from the counterfactual baseline. **(B)** Weekly Relative Change (%): The weekly relative change represents the percentage difference between observed and counterfactual estimates at each time point. Red points and lines indicate temporal variation, and the dashed horizontal line at 0% serves as a reference. This panel is presented for descriptive purposes only to illustrate temporal dynamics relative to the counterfactual trajectory. CrI are not displayed because posterior relative-effect distributions become highly unstable and strongly asymmetric when counterfactual values approach zero, leading to visually uninformative interval expansion. This panel is therefore intended to illustrate temporal dynamics relative to the counterfactual trajectory rather than to provide formal inference.

The cumulative effect analysis indicated that the cumulative observed influenza burden was lower than the projections under the counterfactual scenario over 2020–2025 (Figure 3).

The full-period cumulative relative change (2020–2025) was −70.4%. For descriptive completeness, the posterior distribution of the ratio-based estimand yielded a median of −76.8% with a wide and asymmetric 95% CrI (−90.9% to 233.9%). This interval is not used for inference due to instability of the estimator when the cumulative counterfactual approaches zero in some posterior draws. During the peak intervention period (2020–2022), the cumulative relative effect was −101.1% (95% CrI: −117.0% to −100.5%), indicating a predominantly negative posterior effect during this period.

Annual estimates were −121.6% (2020), −120.7% (2021), −61.6% (2022), −33.6% (2023), and −37.6% (2024), indicating a gradual return toward baseline.

#### 3.2.2 Seasonal epidemic intensity classification across influenza seasons

Overall, observed influenza peak activity declined markedly during the early COVID-19 pandemic and subsequently rebounded in post-pandemic seasons relative to counterfactual estimates (Table 1).

**Table 1:** Comparison of observed and counterfactual peak influenza activity across influenza seasons.

Season	Counterfactual Peak, median (95% CrI)	Observed Peak	Difference, median (95% CrI)
2019/2020	5.54 (4.41, 7.54)	5.37	−0.17 (−2.17, 0.95)
2020/2021	5.76 (0.34, 11.73)	−0.35	−6.11 (−12.08, −0.69)
2021/2022	5.62 (−1.96, 13.87)	2.66	−2.96 (−11.21, 4.62)
2022/2023	5.78 (−4.01, 15.86)	7.33	1.55 (−8.54, 11.34)
2023/2024	5.54 (−5.62, 17.41)	6.47	0.93 (−10.94, 12.09)
2024/2025	5.71 (−7.16, 18.65)	5.92	0.21 (−12.73, 13.07)

CrI, credible interval. Season refers to the winter influenza season defined as epidemiological week 40 of a given year through week 20 of the following year. Counterfactual peak estimates were derived from posterior predictive distributions generated by the BSTS model under a hypothetical no-pandemic scenario. Counterfactual values and peak differences are summarized using posterior medians and 95% CrIs. Posterior uncertainty widened substantially with increasing forecast horizon after 2022, and therefore estimates for 2022/2023–2024/2025 should be interpreted as descriptive projections rather than formal inferential results. Negative differences indicate observed peak activity lower than the counterfactual prediction.

Epidemic intensity thresholds derived using the MEM are provided in Supplementary Material S3. The epidemic threshold was estimated at −0.746, with thresholds for low-, medium-, and high-intensity epidemics of 2.568, 2.955, and 3.095, respectively.

Comparisons between observed and counterfactual peak intensity revealed substantial inter-seasonal variation. During the 2019/2020 season, observed peak activity remained close to the counterfactual estimate.

The greatest suppression occurred during the 2020/2021 season, when observed peak activity was substantially lower than the counterfactual prediction (difference: −5.57, 95% CrI: −12.08 to −0.69), corresponding to a relative reduction of approximately 103%.

During the 2021/2022 season, observed peak activity also remained below the counterfactual estimate (difference: −2.52, 95% CrI: −11.21 to 4.62), although the credible interval overlapped zero.

From the 2022/2023 season onward, observed peak activity exceeded the corresponding counterfactual estimates. However, posterior uncertainty widened substantially during later seasons, and these post-2022 comparisons should therefore be interpreted descriptively rather than inferentially.

#### 3.2.3 Temporal structure of influenza epidemics based on the modified MCM

Overall, influenza epidemic duration was consistently shortened during the COVID-19 pandemic period, mainly driven by earlier end, while changes in onset and peak timing were heterogeneous across seasons (Table 2).

**Table 2:** Comparison of observed and counterfactual temporal characteristics of influenza activity based on modified MCM.

Season	Onset (Obs)	Onset (CF)	Δ Onset	End (Obs)	End (CF)	Δ End	Duration (Obs)	Duration (CF)	Δ Duration	Peak Week (Obs/CF)
2019/2020	6	6	0	22	30	−8	17	25	−8	15/15
2020/2021	2	10	−8	13	29	−16	12	20	−8	11/14
2021/2022	6	9	−3	29	29	0	24	21	+3	21/14
2022/2023	18	9	+9	30	29	+1	13	21	−8	25/14
2023/2024	5	9	−4	19	29	−10	15	21	−6	14/14
2024/2025	9	9	0	31	29	+2	23	21	+2	15/14

All weeks are reported as relative week indices within each influenza season, with week 1 corresponding to epidemiological week 40 of the surveillance year. Δ represents the difference between observed and counterfactual estimates (Observed – Counterfactual). Obs, Observed; CF, Counterfactual; MCM, Maximum Curvature Method. Season refers to the winter influenza season defined as epidemiological week 40 of a given year through week 20 of the following year.

Temporal characteristics of influenza epidemics were evaluated using the modified MCM (Table 2).

Changes in epidemic onset varied across seasons. Earlier onset occurred in several seasons, including 2020/2021, 2021/2022 and 2023/2024, whereas delayed onset occurred in 2022/2023. In several seasons, such as 2019/2020 and 2024/2025, onset remained broadly consistent with counterfactual estimates.

Changes in epidemic end were more consistent across seasons. During the early pandemic seasons, particularly 2019/2020 and 2020/2021, end occurred earlier than counterfactual projections, resulting in a shortened epidemic tail.

Consequently, epidemic duration was generally shorter during pandemic seasons, primarily driven by earlier end rather than delayed onset. In contrast, peak timing showed minimal deviation from counterfactual estimates overall, although delayed and advanced peaks were observed across seasons, including during the pandemic and transition period.

## 4 Discussion

Using long-term surveillance data and a counterfactual modeling framework, this study quantified the indirect impact of the COVID-19 pandemic on winter influenza activity in Wuxi, China. The cumulative relative effect for the full period 2020–2025 yielded a descriptive point estimate of −70.4%, indicating an overall reduction relative to the counterfactual scenario. However, posterior uncertainty increased progressively during the extended post-pandemic forecasting period, particularly after 2022. Therefore, this full-period estimate should be interpreted cautiously as a descriptive summary rather than a robust inferential effect estimate. In contrast, inferential interpretation primarily focused on the peak intervention period (2020–2022), during which the cumulative relative effect was −101.1% (95% CrI: −117.0% to −100.5%), indicating consistently negative posterior effects across all draws. The suppression was most pronounced during the early pandemic phase, when influenza transmission was nearly interrupted. Although influenza activity re-emerged following the relaxation of NPIs, recovery patterns were heterogeneous across seasons and did not represent a simple return to pre-pandemic dynamics.

The dramatic reduction in influenza activity during the 2020/2021 season is consistent with observations reported in Europe, North America, Australia, and East Asia during periods of stringent NPIs (8, 13, 25, 26). Measures including mask mandates, mobility restrictions, school closures, and enhanced hygiene practices likely reduced opportunities for respiratory virus transmission (9, 27). International travel restrictions may also have disrupted viral importation and regional dissemination pathways (28, 29). Changes in healthcare-seeking behavior and diagnostic practices during the pandemic may also have contributed to reduced case detection (30). Together, these factors likely contributed to the marked reduction in influenza activity observed during periods of intensive COVID-19 control. As public health measures were gradually relaxed and population mobility resumed, influenza activity re-emerged in many settings (31); however, several studies have reported atypical post-pandemic seasons characterized by delayed onset (11), altered peak timing (32), or unusually high epidemic intensity (27, 32, 33).

After the relaxation of NPIs, influenza resurgence exhibited pronounced inter-seasonal heterogeneity: peak magnitude was lower than counterfactual estimates during the early pandemic period and exceeded counterfactual projections in subsequent seasons. The observation that epidemic activity exceeded counterfactual projections in certain post-pandemic seasons may be explained by several mechanisms. A primary explanation is the accumulation of an “immunity gap” resulting from the prolonged suppression of influenza circulation during the COVID-19 pandemic, which likely increased population susceptibility and facilitated larger outbreaks upon virus reintroduction. Additionally, the resumption of population mobility and the re-establishment of transmission networks may have accelerated epidemic spread. Furthermore, viral evolutionary processes, such as antigenic drift during periods of reduced circulation, may have contributed to enhanced transmissibility or immune escape. Collectively, these factors may explain the temporary exceedance of influenza activity over expected baseline levels. Beyond changes in epidemic magnitude, we identified perturbations in the temporal structure of influenza epidemics. Whereas conventional surveillance analyses often focus primarily on epidemic intensity, the modified MCM-based temporal framework used here revealed shifts in epidemic onset, duration, and end relative to counterfactual projections. These descriptive findings suggest that post-pandemic influenza dynamics involved changes in both epidemic magnitude and timing.

Notably, even in seasons where peak intensity partially recovered, deviations in epidemic timing persisted, indicating that influenza transmission dynamics may have undergone structural reorganization rather than simple magnitude modulation. Several mechanisms discussed in previous studies may help explain these temporal anomalies. First, prolonged suppression of influenza circulation may have led to the accumulation of susceptible individuals (27), potentially altering both the timing and magnitude of subsequent outbreaks. Second, disruptions to viral ecological dynamics, which include changes in competition and co-circulation among respiratory viruses (9, 34, 35), may have reshaped seasonal transmission patterns. Third, persistent behavioral changes, such as modified social contact patterns and continued precautionary practices (27, 36), may have influenced transmission dynamics even after formal policy relaxation. Taken together, these mechanisms indicate that the observed temporal perturbations likely reflect complex and interacting drivers rather than a single dominant factor.

From a methodological perspective, this study combines composite surveillance indicators with BSTS modeling (37) to construct an explicit counterfactual baseline, enabling the quantification of pandemic-associated deviations from expected influenza dynamics. By integrating MEM (38)-based epidemic intensity classification with temporal metrics derived from the modified MCM, the analytical framework captures both the magnitude and timing of influenza epidemics, providing a more comprehensive characterization of pandemic-associated disruptions than analyses based solely on cases or positivity rate. From a public health perspective, the findings suggest that large-scale interventions can influence not only the intensity but also the temporal structure of seasonal influenza epidemics. The heterogeneous rebound observed after the relaxation of NPIs further suggests that surveillance systems relying exclusively on historical baselines may be insufficient for anticipating atypical epidemic patterns. In the evolving post-pandemic context, influenza monitoring systems may therefore benefit from more adaptive analytical approaches capable of accommodating shifts in population immunity, behavioral patterns, and viral ecological dynamics. Counterfactual inference frameworks such as the one applied in this study may help public health authorities detect anomalous seasons earlier and improve preparedness under conditions of heightened epidemiological uncertainty.

From a public health perspective, the analytical framework used in this study may provide complementary information on epidemic intensity and temporal dynamics for influenza surveillance under changing epidemiological conditions. Counterfactual deviations and changes in epidemic temporal structure may help characterize departures from counterfactual seasonal trajectories. These implications should be interpreted as methodological extensions for future surveillance research rather than components of a real-time operational system.

Several limitations should be noted. First, the composite influenza activity index was constructed using only two surveillance indicators, which may not fully capture the multidimensional nature of influenza transmission dynamics. Although these indicators are consistently available and epidemiologically informative, the exclusion of additional dimensions may limit the comprehensiveness of the measurement framework. Second, the counterfactual model did not explicitly incorporate time-varying covariates, including meteorological conditions, influenza vaccination coverage, population mobility, school calendar effects, and healthcare-seeking behavior, which may affect influenza transmission and introduce residual confounding. For instance, reduced vaccination uptake or delayed immunization campaigns during the pandemic could have heightened population susceptibility in subsequent seasons. Furthermore, alterations in mobility and contact patterns likely modulated transmission intensity. Additionally, uncertainty in the BSTS-based counterfactual projections increased progressively during the extended post-pandemic forecasting period, leading to substantially wider CrIs after 2022. Consequently, estimates for later post-pandemic seasons should be interpreted cautiously and primarily as non-inferential forecasts rather than precise causal effect estimates. Third, the surveillance data may be subject to reporting bias and fluctuations in healthcare utilization, both of which were significantly disrupted during the COVID-19 pandemic. Nevertheless, Wuxi benefits from relatively strong diagnostic capacity and healthcare accessibility, and the use of a composite surveillance index was intended to mitigate biases arising from fluctuations in testing intensity or healthcare utilization. Finally, as an observational analysis, the findings primarily reflect associations rather than definitive causal effects.

In conclusion, this study provides quantitative evidence that the COVID-19 pandemic substantially suppressed influenza activity and was associated with heterogeneous alterations in epidemic temporal structure across post-pandemic seasons. These results underscore that post-pandemic influenza dynamics cannot be assumed to automatically revert to historical norms. Integrating counterfactual modeling with multidimensional epidemic metrics provides a useful framework for monitoring and interpreting seasonal respiratory virus activity in an evolving epidemiological landscape.

## Data Availability

The data analyzed in this study is subject to the following licenses/restrictions: The dataset is owned by the Chinese Center for Disease Control and Prevention and the Wuxi Influenza Surveillance Network. It is not publicly available due to institutional and privacy restrictions. Researchers may request access by contacting the corresponding author with a reasonable research proposal, subject to approval by the data owners. Requests to access these datasets should be directed to Yuan Shen,wxcdcshy@163.com.

## References

[1] Iuliano AD Roguski KM Chang HH Muscatello DJ Palekar R Tempia S . Estimates of global seasonal influenza-associated respiratory mortality: a modelling study. Lancet (2018) 391:1285-1300. doi: 10.1016/s0140-6736(17)33293-2 29248255 PMC5935243

[2] Neumann G Kawaoka Y. Seasonality of influenza and other respiratory viruses. EMBO Mol Med (2022) 14:e15352. doi: 10.15252/emmm.202115352 PMC8988196 35157360

[3] He M Wang Y Ding X Gao Y Shi C. Impact of meteorological factors on influenza incidence in Wuxi from 2014 to 2019: a time series and comprehensive analysis. Front Public Health (2025) 13:1656111. doi: 10.3389/fpubh.2025.1656111 PMC12370636 40860532

[4] Shu YL Fang LQ de Vlas SJ Gao Y Richardus JH Cao WC. Dual seasonal patterns for influenza, China. Emerg Infect Dis (2010) 16:725-726. doi: 10.3201/eid1604.091578 20350403 PMC3321959

[5] Si X Wang L Mengersen K Hu W. Epidemiological features of seasonal influenza transmission among 11 climate zones in Chinese Mainland. Infect Dis Poverty (2024) 13:4. doi: 10.1186/s40249-024-01173-9 PMC10777546 38200542

[6] Cai J Wu Y Liu H Deng Z Yi L Lai L . China's post-zero-COVID Omicron wave: a Bayesian analysis. Proc Natl Acad Sci U S A (2025) 122:e2514157122. doi: 10.1073/pnas.2514157122 41289400 PMC12685082

[7] Goldberg EE Lin Q Romero-Severson EO Ke R. Swift and extensive Omicron outbreak in China after sudden exit from ‘zero-COVID' policy. Nat Commun (2023) 14:3888. doi: 10.1038/s41467-023-39638-4 PMC10314942 37393346

[8] Feng L Zhang T Wang Q Xie Y Peng Z Zheng J . Impact of COVID-19 outbreaks and interventions on influenza in China and the United States. Nat Commun (2021) 12:3249. doi: 10.1038/s41467-021-23440-1 34059675 PMC8167168

[9] Olsen SJ Winn AK Budd AP Prill MM Steel J Midgley CM . Changes in influenza and other respiratory virus activity during the COVID-19 pandemic-United States, 2020-2021. Am J Transplant (2021) 21:3481-3486. doi: 10.1111/ajt.16049 34624182 PMC8653380

[10] Zhao C Zhang T Guo L Sun S Miao Y Yung CF . Characterising the asynchronous resurgence of common respiratory viruses following the COVID-19 pandemic. Nat Commun (2025) 16:1610. doi: 10.1038/s41467-025-56776-z PMC11825952 39948338

[11] Chen X Chen H Tao F Chen Y Zhou Y Cheng J . Global analysis of influenza epidemic characteristics in the first two seasons after lifting the nonpharmaceutical interventions for COVID-19. Int J Infect Dis (2025) 151:107372. doi: 10.1016/j.ijid.2024.107372 39710136

[12] Fricke LM Glöckner S Dreier M Lange B. Impact of non-pharmaceutical interventions targeted at COVID-19 pandemic on influenza burden - a systematic review. J Infect (2021) 82:1-35. doi: 10.1016/j.jinf.2020.11.039 PMC9183207 33278399

[13] Olsen SJ Azziz-Baumgartner E Budd AP Brammer L Sullivan S Pineda RF . Decreased influenza activity during the COVID-19 pandemic - United States, Australia, Chile, and South Africa, 2020. MMWR Morb Mortal Wkly Rep (2020) 69:1305-1309. doi: 10.15585/mmwr.mm6937a6 PMC7498167 32941415

[14] Mohtashemi M Kleinman K Yih WK. Multi-syndrome analysis of time series using PCA: a new concept for outbreak investigation. Stat Med (2007) 26:5203-5224. doi: 10.1002/sim.2872 17476653

[15] Delecroix C Ten Bosch Q Van Nes EH van de Leemput IA. Multivariate resilience indicators to anticipate vector-borne disease outbreaks: a West Nile virus case-study. PLoS Comput Biol (2025) 21:e1012703. doi: 10.1371/journal.pcbi.1012703 41082562 PMC12539738

[16] Song S Li Q Shen L Sun M Yang Z Wang N . From outbreak to near disappearance: how did non-pharmaceutical interventions against COVID-19 affect the transmission of influenza virus? Front Public Health (2022) 10:863522. doi: 10.3389/fpubh.2022.863522 35425738 PMC9001955

[17] Xiao J Dai J Hu J Liu T Gong D Li X . Co-benefits of nonpharmaceutical intervention against COVID-19 on infectious diseases in China: a large population-based observational study. Lancet Reg Health West Pac (2021) 17:100282. doi: 10.1016/j.lanwpc.2021.100282 PMC8484818 34611630

[18] Zhang X Yang L Chen T Wang Q Yang J Zhang T . Predicting influenza-like illness trends based on sentinel surveillance data in China from 2011 to 2019: a modelling and comparative study. Infect Dis Model (2024) 9:816-827. doi: 10.1016/j.idm.2024.04.010 38725432 PMC11079460

[19] Kang M Tan X Ye M Liao Y Song T Tang S. The moving epidemic method applied to influenza surveillance in Guangdong, China. Int J Infect Dis (2021) 104:594-600. doi: 10.1016/j.ijid.2021.01.058 33515775

[20] Kramer SC Shaman J. Development and validation of influenza forecasting for 64 temperate and tropical countries. PLoS Comput Biol (2019) 15:e1006742. doi: 10.1371/journal.pcbi.1006742 PMC6411231 30811396

[21] Feroze N. Forecasting the patterns of COVID-19 and causal impacts of lockdown in top five affected countries using Bayesian Structural Time Series Models. Chaos Solitons & Fractals (2020) 140:110196. doi: 10.1016/j.chaos.2020.110196 32834662 PMC7420989

[22] Brett TS Rohani P. Collateral effects of COVID-19 pandemic control on the US infectious disease landscape. Science (2025) 390:510-515. doi: 10.1126/science.adw4964 41166479

[23] Rakocevic B Grgurevic A Trajkovic G Mugosa B Sipetic Grujicic S Medenica S . Influenza surveillance: determining the epidemic threshold for influenza by using the Moving Epidemic Method (MEM), Montenegro, 2010/11 to 2017/18 influenza seasons. Euro Surveill (2019) 24:1800042. doi: 10.2807/1560-7917.Es.2019.24.12.1800042 PMC6440585 30914080

[24] Cai J Zhang B Xu B Chan KKY Chowell G Tian H . A maximum curvature method for estimating epidemic onset of seasonal influenza in Japan. BMC Infect Dis (2019) 19:181. doi: 10.1186/s12879-019-3777-x PMC6383251 30786869

[25] Sullivan SG Carlson S Cheng AC Chilver MB Dwyer DE Irwin M . Where has all the influenza gone? The impact of COVID-19 on the circulation of influenza and other respiratory viruses, Australia, March to September 2020. Euro Surveill (2020) 25:2001847. doi: 10.2807/1560-7917.Es.2020.25.47.2001847 PMC7693168 33243355

[26] Huang QS Wood T Jelley L Jennings T Jefferies S Daniells K . Impact of the COVID-19 nonpharmaceutical interventions on influenza and other respiratory viral infections in New Zealand. Nat Commun (2021) 12:1001. doi: 10.1038/s41467-021-21157-9 PMC7881137 33579926

[27] Baker RE Park SW Yang W Vecchi GA Metcalf CJE Grenfell BT. The impact of COVID-19 nonpharmaceutical interventions on the future dynamics of endemic infections. Proc Natl Acad Sci U S A (2020) 117:30547-30553. doi: 10.1073/pnas.2013182117 PMC7720203 33168723

[28] Shi S Tanaka S Ueno R Gilmour S Tanoue Y Kawashima T . Travel restrictions and SARS-CoV-2 transmission: an effective distance approach to estimate impact. Bull World Health Organ (2020) 98:518-529. doi: 10.2471/BLT.20.255679 32773897 PMC7411317

[29] Yang B Sullivan SG Du Z Tsang TK Cowling BJ. Effectiveness of international travel controls for delaying local outbreaks of COVID-19. Emerg Infect Dis (2022) 28:251-253. doi: 10.3201/eid2801.211944 34647863 PMC8714230

[30] Moynihan R Sanders S Michaleff ZA Scott AM Clark J To EJ . Impact of COVID-19 pandemic on utilisation of healthcare services: a systematic review. BMJ Open (2021) 11:e045343. doi: 10.1136/bmjopen-2020-045343 PMC7969768 33727273

[31] Eden J-S Sikazwe C Xie R Deng Y-M Sullivan SG Michie A . Off-season RSV epidemics in Australia after easing of COVID-19 restrictions. Nat Commun (2022) 13:2884. doi: 10.1038/s41467-022-30485-3 PMC9130497 35610217

[32] Zhang L Duan W Ma C Zhang J Sun Y Ma J . An intense out-of-season rebound of influenza activity after the relaxation of coronavirus disease 2019 restrictions in Beijing, China. Open Forum Infect Dis (2024) 11:ofae163. doi: 10.1093/ofid/ofae163 PMC10995958 38585185

[33] Chen D Zhang T Chen S Ru X Shao Q Ye Q . The effect of nonpharmaceutical interventions on influenza virus transmission. Front Public Health (2024) 12:1336077. doi: 10.3389/fpubh.2024.1336077 PMC10881707 38389947

[34] Nickbakhsh S Mair C Matthews L Reeve R Johnson PCD Thorburn F . Virus-virus interactions impact the population dynamics of influenza and the common cold. Proc Natl Acad Sci U S A (2019) 116:27142-27150. doi: 10.1073/pnas.1911083116 PMC6936719 31843887

[35] Kramer SC Pirikahu S Casalegno J-S Domenech de Cellès M. Characterizing the interactions between influenza and respiratory syncytial viruses and their implications for epidemic control. Nat Commun (2024) 15:10066. doi: 10.1038/s41467-024-53872-4 PMC11579344 39567519

[36] Barr I. Influenza in Australia before, during and after the COVID-19 pandemic. Microbiology Australia (2024) 45:MA24052. doi: 10.1071/MA24052

[37] Brodersen KH Gallusser F Koehler J Remy N Scott SL. Inferring causal impact using Bayesian structural time-series models. Ann Appl stat (2015) 9(1):247-274. doi: 10.1214/14-AOAS788

[38] Vega T Lozano JE Meerhoff T Snacken R Mott J Ortiz de Lejarazu R . Influenza surveillance in Europe: establishing epidemic thresholds by the moving epidemic method. Influenza Other Respir Viruses (2013) 7:546-558. doi: 10.1111/j.1750-2659.2012.00422.x PMC5855152 22897919

