# Supplementary material for: Disruption of winter influenza activity in Wuxi, China during and after the COVID-19 pandemic (2013–2025): a counterfactual time-series analysis
Source: Front Public Health. 2026 Jun 18;14:1851342. doi: 10.3389/fpubh.2026.1851342 (PMC13323241; doi:10.3389/fpubh.2026.1851342)
Supplement: Supplementary file 1 [file Table_1.docx]

Supplementary Materials

# Supplementary Methods

## **S1. Construction of the composite influenza activity index**

The composite influenza activity index at week *t* was defined as the first principal component derived from two standardized indicators: log-transformed influenza cases and smoothed influenza positivity proportion. Specifically:

$\boldsymbol{I}_{\boldsymbol{t}}\boldsymbol{=}\boldsymbol{w}_{\boldsymbol{1}}$**·**$\boldsymbol{z}_{\boldsymbol{1}}\boldsymbol{(t)}$**+**$\boldsymbol{w}_{\boldsymbol{2}}$**·**$\boldsymbol{z}_{\boldsymbol{2}}\boldsymbol{(t)}$

where:

​ $\boldsymbol{z}_{\boldsymbol{1}}\boldsymbol{(t)=}\frac{\boldsymbol{log(1+}\boldsymbol{cases}_{\boldsymbol{t}}\boldsymbol{)-}\boldsymbol{\mu}_{\boldsymbol{1}}}{\boldsymbol{\sigma}_{\boldsymbol{1}}}$

**​** $\boldsymbol{z}_{\boldsymbol{2}}\boldsymbol{(t)=}\frac{\frac{\boldsymbol{1}}{\boldsymbol{5}}\sum_{\boldsymbol{k=-2}}^{\boldsymbol{2}} \boldsymbol{positivity}_{\boldsymbol{t+k}}\boldsymbol{-}\boldsymbol{\mu}_{\boldsymbol{2}}}{\boldsymbol{\sigma}_{\boldsymbol{2}}}$

Here, $\mu_{1}$, $\sigma_{1}$ and $\mu_{2}$, $\sigma_{2}$ denote the mean and standard deviation of each variable estimated from the pre-pandemic training period.

The weights $w_{1}$ and $w_{2}$ correspond to the loadings of the first principal component obtained from PCA. In this study, the first principal component explained the majority of variance and was therefore used as the composite influenza activity index.

To ensure interpretability, the sign of the component was adjusted such that higher values consistently indicate higher influenza activity.

## **S2. Identification of Epidemic Temporal Structure Using a Modified Maximum Curvature Method**

To systematically characterize the temporal structure of seasonal influenza epidemics and ensure comparability across seasons, we implemented a modified MCM applied to the weekly composite influenza activity index derived from PCA.

Because the PCA-derived composite index is standardized and dimensionless, it does not correspond directly to absolute cases. Consequently, conventional threshold-based epidemic detection approaches cannot be directly applied. The modified MCM provides a scale-adaptive framework capable of identifying turning points in epidemic trajectories without relying on absolute incidence thresholds.

For each influenza season, the observation window was defined as epidemiological week 40 of year t through week 20 of year *t*+1. Within this window, local curvature of the epidemic trajectory was estimated using a symmetric sliding window of five consecutive weeks. Curvature quantifies the geometric bending of the epidemic curve and thus captures structural changes in transmission dynamics, enabling the detection of points where epidemic growth accelerates or decelerates.

Epidemic onset and termination were identified using two complementary criteria: the magnitude of local curvature and the direction of the epidemic trend. Specifically, epidemic onset was defined as the week with maximum curvature during the ascending phase of the epidemic trajectory, whereas epidemic termination was defined as the week with maximum curvature during the descending phase.

To ensure cross-season comparability and reduce sensitivity to extreme seasonal peaks, candidate onset and termination points were restricted to weeks with index values below a season-specific activity threshold, defined as the 75th percentile of the seasonal composite index distribution.

Epidemic duration was defined as the inclusive interval between the identified onset and termination weeks. Peak timing was defined as the epidemiological week with the maximum composite index value within each influenza season.

Robustness was evaluated through sensitivity analyses that varied both the sliding window length (3–7 weeks) and the seasonal activity threshold (70th–80th percentiles). Temporal indicators were expressed as relative week indices within the standardized influenza season, with week 1 corresponding to epidemiological week 40.

This analytical framework enables objective and scale-invariant identification of epidemic timing, while remaining robust to irregular or interrupted epidemic curves, such as those observed during periods of extensive NPIs implementation.

Algorithm workflow:

1. Extract seasonal observation window (week 40 to week 20 of the following year).
2. Estimate local curvature and trend direction for each week using a symmetric sliding window (default: 5 weeks).
3. Identify candidate epidemic onset and termination based on curvature magnitude and trend direction, restricted to values below the seasonal threshold.
4. Derive epidemic duration and peak timing.
5. Quantify differences between observed and counterfactual temporal indicators.
6. Perform sensitivity analyses across alternative window lengths and threshold percentiles.

## S3. Seasonal Intensity Classification Using the Moving Epidemic Method

Six pre-pandemic influenza seasons (2013/2014–2018/2019) were used as the reference training dataset. Each influenza season was defined as epidemiological week 40 through week 20 of the subsequent year, forming a standardized 33-week winter observation window.

Weekly composite influenza activity index values were aligned across seasons to construct a 33 × 6 seasonal activity matrixes. Missing observations were imputed using linear interpolation combined with forward and backward filling procedures to ensure matrix completeness.

The epidemic threshold was defined as the 40th percentile of the historical pre-pandemic distribution. Seasonal intensity thresholds were then derived from the distribution of historical seasonal peak values: the 50th percentile (low intensity), 90th percentile (medium intensity), and 97.5th percentile (high intensity). Seasonal peaks exceeding the highest threshold were classified as very high intensity.

These thresholds were applied consistently to both observed and counterfactual seasonal peak values, enabling comparative assessment of influenza epidemic intensity patterns during and after the COVID-19 pandemic.

# Supplementary Tables

## Table S1. Population of Wuxi and its administrative districts based on the 2025 Wuxi Statistical Yearbook

This table provides official population statistics for Wuxi and its administrative districts. The data are presented to characterize the demographic background of the study area and to support the assessment of the representativeness of the sentinel surveillance system.

| District | Population (10⁴) |
| --- | --- |
| Liangxi | 98.40 |
| Binhu (incl. EDZ) | 92.67 |
| Xinwu | 73.12 |
| Huishan | 90.29 |
| Xishan | 89.24 |
| Jiangyin | 178.68 |
| Yixing | 128.10 |
| Total | 750.50 |

**Note:** Population data were obtained from the 2025 Wuxi Statistical Yearbook. The Economic Development Zone is administratively included within Binhu District and is therefore reported under Binhu District in this table. These data are provided for demographic context to support interpretation of the spatial distribution of sentinel hospitals and were not used as analytical variables in the statistical models.

## Table S2. MCMC convergence diagnostics and posterior predictive robustness of the BSTS model

This table reports MCMC convergence diagnostics and posterior predictive robustness for the BSTS model under primary and sensitivity specifications.

| Parameter/ comparison | *MCMC* setting | *R̂* | *ESS* | Posterior predictive result |
| --- | --- | --- | --- | --- |
| $\sigma_{obs}$ | Primary | 1.015 | 53.7 |  |
| $\sigma_{level}$ | Primary | 1.011 | 1,167.6 |  |
| $\sigma_{seasonal}$ | Primary | 3.336 | 12.0 |  |
| $\sigma_{obs}$ | Sensitivity | 1.004 | 271.6 |  |
| $\sigma_{level}$ | Sensitivity | 1.000 | 6,213.7 |  |
| $\sigma_{seasonal}$ | Sensitivity | 1.291 | 25.6 |  |
| Posterior predictive |  |  |  | Correlation = 0.9999;  *MAE* in-sample = 0.014;  *MAE* forecast = 0.040 |

**Note**: *R̂* values are computed using split-chain Gelman–Rubin diagnostics and are commonly interpreted as indicating approximate convergence when *R̂* < 1.1. Effective sample size (*ESS*) reflects the amount of independent information in the posterior samples, with higher values indicating better mixing.

The primary *MCMC* specification uses 4,000 iterations with 1,000 burn-in, while the sensitivity analysis uses 20,000 iterations with 5,000 burn-in to assess robustness to chain length.

The seasonal variance parameter shows consistently elevated *R̂* values and low *ESS* across both specifications, suggesting weak identifiability due to partial confounding between the seasonal and local level components in the state-space decomposition.

Despite this, posterior predictive comparisons (in-sample and forecast periods) show near-perfect agreement between specifications (correlation = 0.9999), indicating that substantive conclusions are robust to *MCMC* specification and parameter-level non-identifiability.

## Table S3. Sensitivity analyses of BSTS model specifications for influenza counterfactual estimatio

Sensitivity analyses of Bayesian structural time series (BSTS) model specifications used to evaluate the robustness of counterfactual estimates of influenza activity in the absence of COVID-19. Alternative specifications include variations in seasonal structure, training period length, and error dynamics.

| Model specification | Seasonal component | Training period | Pandemic estimate  (% change) |
| --- | --- | --- | --- |
| Main model | 52-week seasonal + local level | 2013–2019 | -38.3% |
| Sensitivity 1 | 12-week seasonal + local level | 2013–2019 | -29.9% |
| Sensitivity 2 | 52-week seasonal + local level | 2015–2019 | -38.5% |
| Sensitivity 3 | 52-week seasonal + local level + AR(1) | 2013–2019 | -30.2% |

**Notes:** The pandemic period was defined as influenza seasons 2020/2021 to 2022/2023, corresponding to sustained COVID-19-related non-pharmaceutical interventions and altered respiratory virus transmission dynamics.

The post-pandemic period (2023/2024–2024/2025) was not included in the estimation of pandemic impact but was retained for descriptive comparison of recovery dynamics.

All estimates represent posterior mean percentage change between observed influenza activity and BSTS-derived counterfactual predictions during the defined pandemic period.

Counterfactual estimates were generated using Bayesian structural time series models implemented with Markov Chain Monte Carlo (MCMC) sampling.

## Table S4. Sensitivity analysis of posterior inference, counterfactual stability, and MCMC thinning for BSTS model

This table summarizes posterior sampling adequacy, robustness of counterfactual trajectories, and sensitivity of cumulative effect estimates under alternative MCMC and thinning specifications.

| Category | Metric/ Specification | Result |
| --- | --- | --- |
| Posterior sampling | Effective sample size (*ESS*), observation variance | 199.9 |
| Counterfactual robustness | Pearson correlation (main vs AR(1) posterior mean trajectories) | 0.978 |
| Causal effect (2020–2022) | Main model, median (95% CrI) | −100.2% (−101.9%, −98.3%) |
| Causal effect (2020–2022) | AR(1) model, median (95% CrI) | −100.2% (−102.5%, −97.4%) |
| Sampling robustness | Thinning sensitivity (*k* = 5, 10), median range | −100.2% (variation < 0.2%) |

**Note**: Effective sample size (*ESS*) was computed for posterior draws of the observation variance parameter, with higher values indicating improved mixing of the MCMC chains. Counterfactual robustness was assessed using Pearson correlation between posterior mean trajectories from the main BSTS specification and an alternative AR(1)-augmented model. Thinning sensitivity was evaluated by subsampling posterior draws at intervals of 5 and 10, showing negligible variation in posterior summaries (<0.2%), indicating stability of causal estimates under different sampling densities.

## Table S5. Sensitivity analysis of epidemic timing estimates

To evaluate the robustness of the modified MCM to parameter selection, we calculated the standard deviation (*SD*) and coefficient of variation (*CV*) of epidemic onset, termination, and duration estimates across combinations of sliding window lengths (3–7 weeks) and seasonal threshold percentiles (70–80%).

Lower *SD* and *CV* values indicate greater stability of the estimated temporal indicators under varying parameter configurations.

| Season | Onset *SD* (weeks) | End *SD* (weeks) | Duration *SD* (weeks) | Onset *CV* (%) | End *CV*(%) | Duration *CV* (%) |
| --- | --- | --- | --- | --- | --- | --- |
| 2019/2020 | 1.41 | 3.38 | 3.34 | 19.7 | 14.3 | 19.1 |
| 2020/2021 | 2.32 | 3.17 | 2.96 | 89.4 | 20.0 | 20.8 |
| 2021/2022 | 4.31 | 0.655 | 4.41 | 52.6 | 2.26 | 20.2 |
| 2022/2023 | 5.43 | 0.492 | 5.25 | 36.2 | 1.66 | 33.5 |
| 2023/2024 | 2.72 | 3.62 | 5.06 | 44.2 | 17.2 | 32.0 |

**Note**: *SD*, standard deviation; *CV*, coefficient of variation. Season refers to the winter influenza season defined as epidemiological week 40 of a given year through week 20 of the following year.

Estimates were obtained from combinations of sliding window lengths (3–7 weeks) and seasonal threshold percentiles (70–80%).

## Table S6. Descriptive Statistics of the Composite Influenza Activity Index

The composite influenza activity index represents the standardized first principal component (*PC1*) derived from multiple surveillance indicators.

| **Period** | ***n*** | ***Mean*** | ***SD*** | ***Minimum*** | ***Maximum*** | ***Median*** | ***IQR*** |
| --- | --- | --- | --- | --- | --- | --- | --- |
| 2013–2019 | 364 | -0.138 | 1.41 | -1.63 | 4.55 | -0.657 | 2.09 |
| 2020–2022 | 157 | -0.010 | 1.77 | -1.63 | 5.37 | -0.807 | 2.99 |
| 2023–2025 | 155 | 2.10 | 2.21 | -1.63 | 7.33 | 1.22 | 3.00 |

**Note:** *SD*, standard deviation; *IQR*, interquartile range.

**Brief description:** Before the COVID-19 pandemic (2013–2019), the composite influenza activity index fluctuated around zero with moderate variability (*SD* = 1.41). During the pandemic period (2020–2022), variability increased slightly (*SD* = 1.77) while the central tendency remained relatively low. In the post-pandemic period (2023–2025), the index showed higher mean values and greater dispersion (*SD* = 2.21) compared with earlier periods.

## Table S7. Model fit and descriptive statistics of the BSTS counterfactual series

The Bayesian structural time series (BSTS) model was trained using pre-pandemic data (2013–2019) to generate counterfactual estimates of influenza activity under a hypothetical scenario without COVID-19-related interventions.

| Category | Statistic | Value |
| --- | --- | --- |
| Model fit (pre-intervention period) | *R²* | 0.868 |
|  | *RMSE* | 0.510 |
|  | *MAE* | 0.377 |
| Counterfactual projection  (2020-2025; *n* = 312 weeks) | *Mean* | 3.462 |
|  | *SD* | 0.711 |
|  | *Minimum* | 2.470 |
|  | *Maximum* | 5.221 |
| Annual counterfactual means | 2020 | 3.51 |
|  | 2021 | 3.52 |
|  | 2022 | 3.52 |
|  | 2023 | 3.51 |
|  | 2024 | 3.37 |
|  | 2025 | 3.37 |

**Note:** *RMSE*, root mean square error; *MAE*, mean absolute error; *SD*, standard deviation.

Counterfactual weekly estimates generated by the BSTS model were aggregated to annual mean values.

**Brief description:** The BSTS model showed a high level of fit during the pre-intervention period (*R²* = 0.868), with low prediction errors (*RMSE* = 0.510; *MAE* = 0.377). Counterfactual projections for 2020–2025 indicated relatively stable expected influenza activity, with annual mean values remaining around 3.5.
